# Association of lipoprotein(a) with left ventricular hypertrophy assessed by electrocardiogram in adults: a large cross-sectional study

**DOI:** 10.3389/fendo.2023.1260050

**Published:** 2023-11-30

**Authors:** Xuejiao Yan, Jing Gong, Zhenwei Wang, Fangfang Wang, Chunjian Qi

**Affiliations:** ^1^ Department of Cardiology, The Affiliated Changzhou No.2 People’s Hospital of Nanjing Medical University, Changzhou, Jiangsu, China; ^2^ Department of Geriatrics, Nanjing Tongren Hospital, School of Medicine, Southeast University, Nanjing, China; ^3^ Department of Cardiology, The First Affiliated Hospital of Zhengzhou University, Zhengzhou, China; ^4^ Medical Research Center, The Affiliated Changzhou No.2 People’s Hospital of Nanjing Medical University, Changzhou, Jiangsu, China

**Keywords:** lipoprotein(a), left ventricular hypertrophy, left ventricular mass index, cardiovascular disease, general population

## Abstract

**Background and aims:**

Increasing evidence supports a causal relationship between lipoprotein(a) [Lp(a)] and atherosclerotic cardiovascular disease, yet its association with left ventricular hypertrophy (LVH) assessed by electrocardiogram (ECG) remains unknown. The aim of this study was to explore the relationship between Lp(a) and LVH assessed by ECG in general population.

**Methods and results:**

In this cross-sectional study, we screened 4,052 adults from the participants of the third National Health and Nutrition Examination Survey for analysis. Lp(a) was regarded as an exposure variable. LVH defined by the left ventricular mass index estimated from ECG was considered as an outcome variable. Multivariate logistic regression and restricted cubic spline (RCS) were used to assess the relationship between Lp(a) and LVH. Individuals with LVH had higher Lp(a) compared to individuals without LVH (P< 0.001). In the fully adjusted model, Lp(a) was strongly associated with LVH when as a continuous variable (per 1-unit increment, OR: 1.366, 95% CI: 1.043-1.789, P = 0.024), and higher Lp(a) remained independently associated with a higher risk of LVH when participants were divided into four groups according to quartiles of Lp(a) (Q4 vs Q1, OR: 1.508, 95% CI: 1.185-1.918, P = 0.001). And in subgroup analysis, this association remained significant among participants< 60 years, ≥ 60 years, male, with body mass index< 30 kg/m^2^, with hypertension and without diabetes (P< 0.05). In addition, we did not observe a nonlinear and threshold effect of Lp(a) with LVH in the RCS analysis (P for nonlinearity = 0.113).

**Conclusion:**

Lp(a) was closely associated with LVH assessed by ECG in general population.

## Introduction

Left ventricular hypertrophy (LVH) is defined as an increase in left ventricular mass (LVM) that can be secondary to an increase in ventricular wall thickness or chamber size (1). Electrocardiography (ECG), echocardiography and magnetic resonance imaging are currently the main diagnostic tools for the evaluation of LVH. Although ECG is less accurate than the other two diagnostic methods in diagnosing LVH, it is widely used in epidemiological studies because of its low cost, convenience and easy availability. However, LVH detected by either method is strongly associated with a higher risk of cardiovascular disease (CVD) and CVD-related mortality (2–6). Therefore, the 2018 European Society of Hypertension (ESH)/European Society of Cardiology (ESC) clinical practice guideline added LVH as a high-risk factor to the CVD risk assessment system and recommended screening for LVH in high-risk populations as well as early identification and intervention of controllable risk factors for LVH to prevent premature CVD or CVD-related death (7). Although current evidence suggests that the risk factors for LVH are composed of some non-modifiable and modifiable factors, including uncontrollable factors such as age, gender and genetic susceptibility and controllable risk factors such as hypertension, diabetes, chronic kidney disease, metabolic disorders, obesity, lack of exercise or unhealthy diet, other potential risk factors may still exist (1, 8–10).

Lipoprotein(a) [Lp(a)] is a low-density lipoprotein cholesterol-like particle bound to apolipoprotein(a), which can participate in the occurrence and development of CVD by promoting oxidation, inflammation, calcification, thrombosis and atherosclerosis (11). Because circulating Lp(a) levels are largely regulated by genes, its absolute risk threshold has not yet reached unity, but current evidence suggests that higher Lp(a) is associated with a higher risk of CVD (12). In recent years, studies of Lp(a) and CVD have been widely conducted as Lp(a) has gradually gained attention and improved measurement methods have been developed. As a result, a growing number of observational studies have shown that Lp(a) is closely related to coronary heart disease (CHD) (13), stroke (14), calcific aortic valve disease (CAVD) (15), hypertension (16), atrial fibrillation (17) and venous thromboembolism (18), and evidence from genetic studies has also confirmed the causal association of Lp(a) with CVD and CVAD (19, 20). A recent study revealed the relationship between Lp(a) and LVH assessed by echocardiography only in patients with acute myocardial infarction (21), whereas the correlation between Lp(a) and LVH assessed by ECG in the general population remains unknown.

Therefore, in order to fill the gap in this research field and provide reference for formulating early prevention and treatment strategies of LVH under the background of higher Lp(a), the aim of this study was to explore the relationship between Lp(a) and LVH assessed by ECG in the general population.

## Methods

### Study population

**Figure 1:**
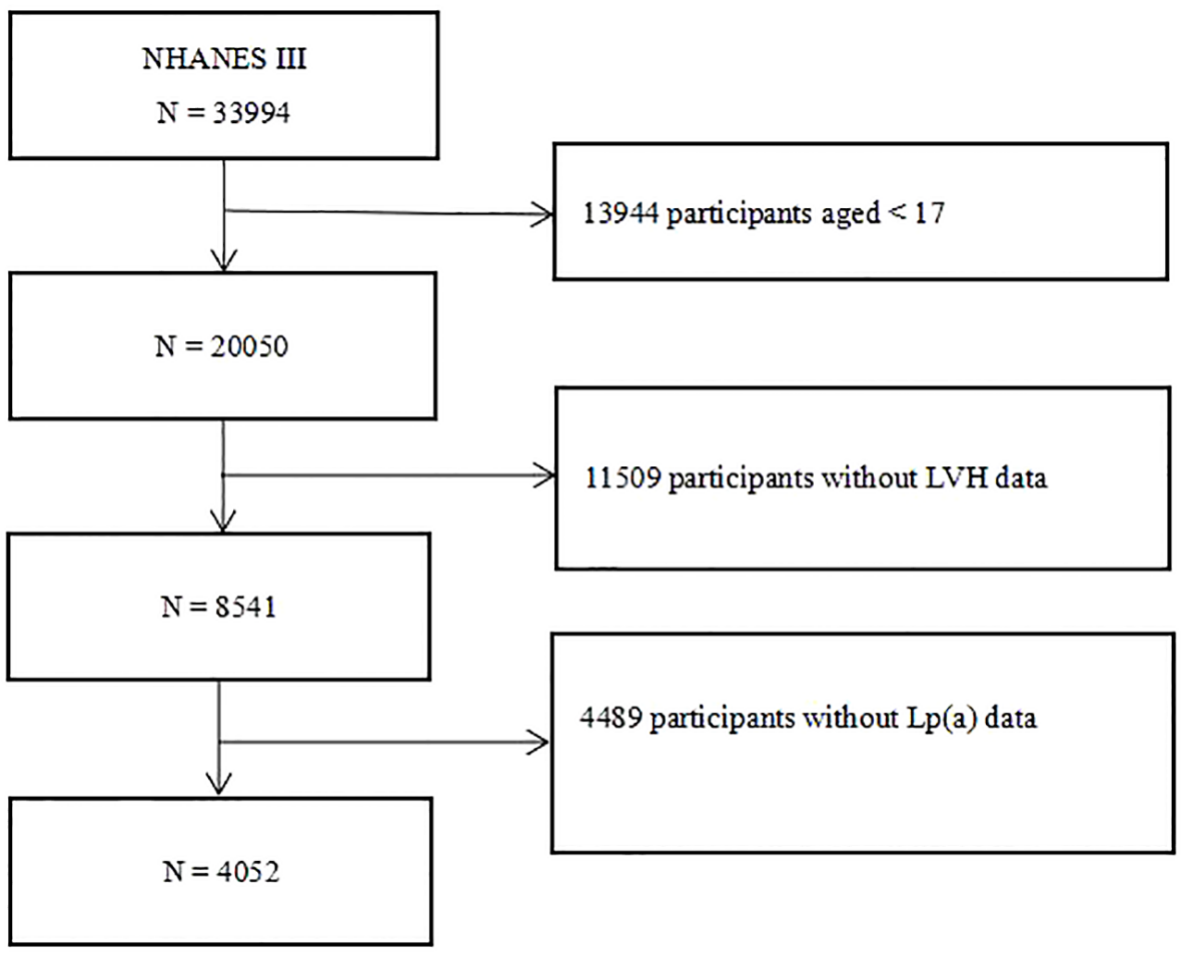
Flow chart of the study participants. NHANES III, the third National Health and Nutrition Examination Survey; Lp(a), lipoprotein(a); LVH, left ventricular hypertrophy.

After excluding individuals younger than 17 years old, without Lp(a) and left ventricular mass, left ventricular mass index (LVMI) or LVH data, we screened 4,052 participants from the third National Health and Nutrition Examination Survey (NHANES III). The flow chart of the study population was shown in **Figure 1**. The NHANES study protocol was approved by the National Center for Health Statistics of the Center for Disease Control and Prevention Institutional Review Board, and all participants signed a written informed consent form when participating in the NHANES. And this study was in line with the Declaration of Helsinki.

### Data collection and definitions

Variables used for analysis in this study included age, sex, race, family poverty income ratio (PIR), ideal exercise, smoking status, drinking, hypertension, diabetes, hypercholesterolemia, hypotensive drugs, hypoglycemic drugs, cholesterol-lowering drugs, body mass index (BMI), systolic blood pressure (SBP), diastolic blood pressure (DBP), triglycerides, total cholesterol (TC), low-density lipoprotein cholesterol (LDL-C), high-density lipoprotein cholesterol (HDL-C), Lp(a), blood urea nitrogen (BUN), creatinine, uric acid (UA), fasting plasma glucose (FPG), hemoglobin A1c (HbA1c), LVM, LVMI, and LVH. All of the above demographic data, comorbidity data, medication data, and biomarker data were obtained using standardized household questionnaires and standard biochemical measurement procedures, the specific methods and contents of which are available on the publicly available NHANES website. In this study, race was divided into four groups: non-Hispanic White, non-Hispanic Black, Mexican-American and Others. Family PIR was divided into three groups: ≤ 1.0, 1.0-3.0, > 3.0. Ideal exercise was defined as ≥ 150 minutes of moderate intensity activity or ≥ 75 minutes of high intensity activity per week. Smoking status was divided into three groups: every day, some days, and not at all. Drinking was defined as drinking at least 12 drinks in a year. Hypertension was defined as pre-existing hypertension, or a mean SBP ≥ 140 mmHg or mean DBP ≥ 90 mmHg when participating in NHANES or taking oral antihypertensive medication. Diabetes was defined as pre-existing diabetes, or FPG ≥ 7.0 mmol/L or HbA1c ≥ 6.5% when participating in NHANES or using hypoglycemic medication. Hypercholesterolemia was defined as having previous hypercholesterolemia.

All participants in this study underwent a 12-lead resting ECG. Trained professionals collected ECG signal data of participants through a Marquette MAC 12 system (Marquette Medical Systems, Milwaukee, Wisconsin), which was then transmitted to an ECG reading centre where the acquired ECG data were coded by Minnesota codes and analyzed by a Novacode ECG measurement, A classification program with an algorithm for classifying ECGs according to Minnesota codes, an algorithm for classifying LVH according to various ECG criteria, and a multivariate statistical model for estimating LVM and LVMI by the routine 12-lead ECG were used (22–28). Specific multivariate linear regression equations for the estimated LVM were shown below (24):

White and black males: LVM=-58.51+0.060QS(III)+0.021R(V5)−0.033QS(V1)−0.296Tp(aVR)+0.316Tn(V6)+1.821QRS.

White female: LVM=134.77+0.023R(V5)−0.155QS(I)+0.070QS(V5)+0.112Tp(V1)−0.123Tp(V6)+0.032R(aVL) .

Black females: LVM=−90.71+0.050R(I)−0.051R(V1)−0.098QS(V6)+0.522Tn(I)+1.848QRS+0.023[R(V6)+QS(V2)] .

LVH is defined as LVMI > 150 g/m^2^ for men or LVMI > 120 g/m^2^ for women. The above reference values of LVMI for LVH defined by sex correspond to the normal upper limits for LVMI based on echocardiographic detection established by the American Society of Echocardiography (23), and these ECG-LVH standards have been proved to have good diagnostic efficacy in a large population-based epidemiological study (24). In addition, we evaluated LVH according to other ECG criteria such as Sokolow-Lyon criterion, Cornell criterion and Cornell product (29, 30).

### Statistical analysis

First, continuous variables that were normally distributed were expressed as mean ± standard deviation and compared between two or four groups using independent samples t-test or one-way ANOVA, respectively, when the variables met both the homogeneity of variance. Continuous variables as non-normally distributed were expressed as median (first quartile, third quartile) and compared between two or four groups using the Mann-Whitney U or Kruskal-Wallis H test, respectively. Categorical variables were expressed as frequencies and percentages, and differences in percentages of categorical variables between groups were assessed using the chi-square or Fisher’s exact test. Second, univariate logistic regression model was used to examine variables associated with LVH (P< 0.05), and these variables were then constructed into four models for multivariate logistic regression analysis of the relationship between Lp(a) and LVH. Model 1 adjusted for age and sex; model 2 adjusted for age, sex, race, family PIR, ideal exercise, and drinking; model 3 adjusted for the variables in model 2 plus diabetes, hypertension, hypoensive drugs, and hypoglycemic drugs; model 4 adjusted the variables in model 3 plus BMI, SBP, DBP, TC, BUN, creatinine, UA, FPG and HbA1c. Subsequently, we assessed the robustness of the relationship between Lp(a) and LVH in five subgroups, including age (< 60 or ≥ 60 years), sex (male or female), BMI (≥ 30 or< 30 kg/m^2^), hypertension (yes or no), and diabetes (yes or no). Third, we explored possible nonlinear relationships and threshold effects of Lp(a) with LVM, LVMI, and LVH using restricted cubic splines (RCS) with three nodes. All statistical methods in this study were performed by SPSS 26.0 (SPSSInc., Chicago, Illinois, USA) and the R programming language (version 4.1.3). A two-tailed P< 0.05 was viewed as statistically significant.

## Results

### Baseline characteristics

First, 4,052 participants (mean age: 60.13 years; 45.80% men) were divided into two groups: non-LVH and LVH groups (**Table 1**). Compared with the non-LVH group, the LVH group had more older people, more women, more non-Hispanic Black, more family PIR of 1.0-3.0, more ideal exercise, more prevalence of hypertension, more prevalence of diabetes, more use of hypotensive drugs, more use of hypoglycemic drugs, fewer drinkers and higher levels of BMI, SBP, DBP, TC, Lp(a), BUN, creatinine, UA, FPG, HbA1c, LVM, and LVMI (P< 0.05). Then, all participants were divided into four groups according to the quartiles of Lp(a): Q1 ≤ 0.04, 0.04< Q2 ≤ 0.17, 0.17< Q3 ≤ 0.36, Q4 > 0.36 (**Table 2**). Age, sex, race, smoking status, drinking, hypertension, diabetes, hypercholesterolemia, hypotensive drugs, hypoglycemic drugs, cholesterol-lowering drugs, DBP, triglycerides, TC, LDL-C, HDL-C, and FPG were statistically significant between these four groups, and the group with higher Lp(a) had higher prevalence of LVH than the group with lower Lp(a) (P< 0.05).

**Table 1:** Baseline characteristics of participants with and without LVH.

Variables	Total population	Non-LVH	LVH	P value
N	4052	3300	752	
Age, years	60.13 ± 13.63	59.05 ± 13.49	64.87 ± 13.24	< 0.001
Sex, male, n (%)	1855 (45.80)	1569 (47.50)	286 (38.00)	< 0.001
Race, n (%)				< 0.001
Non-Hispanic white	1935 (47.80)	1616 (49.00)	319 (42.40)	
Non-Hispanic black	951 (23.50)	707 (21.40)	244 (32.40)	
Mexican-American	966 (23.80)	816 (24.70)	150 (19.90)	
Others	200 (4.90)	161 (4.90)	39 (5.20)	
Family PIR, n (%)				< 0.001
≤ 1.0	762 (20.50)	605 (19.90)	157 (23.10)	
1.0-3.0	1678 (45.10)	1328 (43.70)	350 (51.50)	
> 3.0	1278 (34.40)	1106 (36.40)	172 (25.30)	
Ideal exercise, n (%)				0.029
Yes	2707 (66.80)	2179 (66.10)	528 (70.20)	
No	1344 (33.20)	1120 (33.90)	224 (29.80)	
Smoking status, n (%)				0.362
Every day	1930 (47.60)	1557 (47.20)	373 (49.60)	
Some days	1261 (31.10)	1029 (31.20)	232 (30.90)	
Not at all	861 (21.20)	714 (21.60)	147 (19.50)	
Drinking, n (%)				< 0.001
Yes	1495 (46.70)	1274 (48.30)	221 (39.30)	
No	1703 (53.30)	1361 (51.70)	342 (60.70)	
Comorbidities, n (%)				
Hypertension				< 0.001
Yes	2101 (52.00)	1550 (47.10)	551 (73.40)	
No	1943 (48.00)	1743 (52.90)	200 (26.60)	
Diabetes				< 0.001
Yes	985 (24.30)	750 (22.70)	235 (31.30)	
No	3066 (75.70)	2549 (77.30)	517 (68.80)	
Hypercholesterolemia				0.199
Yes	1111 (40.70)	922 (41.20)	189 (38.10)	
No	1621 (59.30)	1314 (58.80)	307 (61.90)	
Treatment, n (%)				
Hypotensive drugs				< 0.001
Yes	1032 (27.40)	728 (23.80)	304 (42.60)	
No	2735 (72.60)	2325 (76.20)	410 (57.40)	
Hypoglycemic drugs				0.001
Yes	379 (9.40)	284 (8.60)	95 (12.70)	
No	3662 (90.60)	3008 (91.40)	654 (87.30)	
Cholesterol-lowering drugs				0.287
Yes	203 (10.60)	158 (10.20)	45 (12.10)	
No	1712 (89.40)	1386 (89.80)	326 (87.90)	
BMI, kg/m^2^	27.96 ± 5.61	27.86 ± 5.49	28.39 ± 6.11	0.030
SBP, mmHg	133.62 ± 20.24	130.94 ± 18.75	145.38 ± 22.23	< 0.001
DBP, mmHg	76.43 ± 10.45	76.14 ± 9.86	77.69 ± 12.66	0.002
TG, mmol/L	1.50 (1.05, 2.18)	1.49 (1.05, 2.16)	1.55 (1.07, 2.32)	0.057
TC, mmol/L	5.59 ± 1.12	5.56 ± 1.12	5.71 ± 1.11	0.001
LDL−C, mmol/L	3.51 ± 0.99	3.50 ± 0.98	3.57 ± 1.01	0.251
HDL−C, mmol/L	1.31 ± 0.43	1.30 ± 0.42	1.33 ± 0.46	0.083
Lp(a), g/L	0.17 (0.04, 0.36)	0.16 (0.04, 0.35)	0.21 (0.06, 0.47)	< 0.001
BUN, mmol/L	5.48 ± 2.14	5.39 ± 2.07	5.86 ± 2.42	< 0.001
CR, umol/L	100.14 ± 40.91	98.92 ± 34.07	105.52 ± 62.45	0.005
UA, umol/L	328.47 ± 88.99	325.09 ± 87.12	343.35 ± 95.45	< 0.001
FPG, mmol/L	5.39 (5.01, 5.97)	5.37 (5.01, 5.90)	5.53 (5.07, 6.17)	< 0.001
HbA1c, %	5.89 ± 1.33	5.85 ± 1.32	6.05 ± 1.35	< 0.001
LVM, g	156.90 ± 32.21	154.24 ± 29.98	168.53 ± 38.47	< 0.001
LVMI, g/m^2^	106.69 ± 25.15	99.71 ± 18.19	137.07 ± 28.47	< 0.001

Data were expressed as mean ± SD, median (first quartile, third quartile), or n (%). Abbreviation: LVH, left ventricular hypertrophy; PIR, poverty income ratio; BMI, body mass index; SBP, systolic blood pressure; DBP, diastolic blood pressure; TG, triglycerides; TC, total cholesterol; LDL-C, low-density lipoprotein cholesterol; HDL-C, high-density lipoprotein cholesterol; Lp(a), lipoprotein(a); BUN, blood urea nitrogen; CR, creatinine; UA, uric acid; FPG, fasting plasma glucose; HbA1c, hemoglobin A1c; LVM, left ventricular mass; LVMI, left ventricular mass index.

**Table 2:** Baseline characteristics of participants stratified by the quartile of the Lp(a).

	Q1		Q2	Q3	Q4	P value
N	1041		1012	993	1006	
Age, years	61.05 ± 13.47		59.66 ± 13.59	60.29 ± 13.86	59.50 ± 13.59	0.043
Sex, male, n (%)	518 (49.80)		467 (46.10)	460 (46.30)	410 (40.80)	0.001
Race, n (%)						< 0.001
Non-Hispanic white	587 (56.40)		543 (53.70)	435 (43.80)	370 (36.80)	
Non-Hispanic black	62 (6.00)		123 (12.20)	299 (30.10)	467 (46.40)	
Mexican-American	347 (33.30)		283 (28.00)	211 (21.20)	125 (12.40)	
Others	45 (4.30)		63 (6.20)	48 (4.80)	44 (4.40)	
Family PIR, n (%)						0.062
≤ 1.0	201 (21.00)		165 (17.60)	199 (22.00)	197 (21.30)	
1.0-3.0	413 (43.20)		424 (45.30)	406 (45.00)	435 (47.10)	
> 3.0	343 (35.80)		346 (37.00)	298 (33.00)	291 (31.50)	
Ideal exercise, n (%)						0.090
Yes	712 (68.50)		646 (63.80)	661 (66.66)	688 (68.40)	
No	328 (31.50)		366 (36.20)	332 (33.40)	318 (31.60)	
Smoking status, n (%)						< 0.001
Every day	451 (43.30)		493 (48.70)	500 (50.40)	486 (48.30)	
Some days	371 (35.60)		336 (33.20)	274 (27.60)	280 (27.80)	
Not at all	219 (21.00)		183 (18.10)	219 (22.10)	240 (23.90)	
Drinking, n (%)						0.007
Yes	392 (45.80)		400 (52.20)	348 (44.90)	355 (44.40)	
No	464 (54.20)		367 (47.80)	427 (55.10)	445 (55.60)	
Comorbidities, n (%)						
Hypertension						0.002
Yes	521 (50.20)		486 (48.10)	531 (53.60)	563 (56.00)	
No	517 (49.80)		524 (51.90)	460 (46.40)	442 (44.00)	
Diabetes						< 0.001
Yes	318 (30.50)		223 (22.10)	227 (22.90)	217 (21.60)	
No	723 (69.50)		788 (77.90)	766 (77.10)	789 (78.40)	
Hypercholesterolemia						0.003
Yes	270 (37.90)		274 (39.20)	254 (38.90)	313 (46.90)	
No	442 (62.10)		425 (60.80)	399 (61.10)	355 (53.10)	
LVH						0.001
Yes	162 (15.60)		184 (18.20)	182 (18.30)	224 (22.30)	
No	879 (84.40)		828 (81.80)	811 (81.70)	782 (77.70)	
Treatment, n (%)						
Hypotensive drugs						0.001
Yes	246 (25.50)		227 (24.00)	266 (28.80)	293 (31.40)	
No	719 (74.50)		717 (76.00)	659 (71.20)	640 (68.60)	
Hypoglycemic drugs						0.030
Yes	119 (11.50)		95 (9.40)	77 (7.80)	88 (8.80)	
No	917 (88.50)		912 (90.60)	916 (92.20)	917 (91.20)	
Cholesterol-lowering drugs						0.004
Yes	46 (9.20)		50 (10.00)	39 (8.40)	68 (15.10)	
No	456 (90.80)		451 (90.00)	424 (91.60)	381 (84.90)	
BMI, kg/m^2^	28.09 ± 5.31		27.86 ± 5.39	27.91 ± 5.66	27.98 ± 6.08	0.798
SBP, mmHg	133.09 ± 19.44		132.86 ± 19.58	134.04 ± 21.27	134.53 ± 20.63	0.203
DBP, mmHg	75.49 ± 10.15		76.20 ± 10.31	76.90 ± 10.95	77.16 ± 10.32	0.001
TG, mmol/L	1.77 (1.19, 2.65)		1.51 (1.07, 2.18)	1.40 (1.00, 2.00)	1.38 (0.96, 1.92)	< 0.001
TC, mmol/L	5.45 ± 1.16		5.51 ± 1.13	5.57 ± 1.06	5.83 ± 1.10	< 0.001
LDL−C, mmol/L	3.28 ± 1.07		3.44 ± 0.97	3.55 ± 0.92	3.77 ± 0.93	< 0.001
HDL−C, mmol/L	1.25 ± 0.43		1.29 ± 0.41	1.33 ± 0.43	1.36 ± 0.43	< 0.001
BUN, mmol/L	5.53 ± 2.18		5.50 ± 2.16	5.47 ± 2.19	5.40 ± 2.05	0.579
CR, umol/L	99.26 ± 44.17		98.60 ± 42.59	101.45 ± 47.27	101.31 ± 26.30	0.292
UA, umol/L	333.42 ± 90.68		322.57 ± 86.96	328.57 ± 87.38	329.20 ± 90.61	0.053
FPG, mmol/L	5.46 (5.06, 6.21)		5.40 (5.02, 5.88)	5.37 (5.02, 5.94)	5.33 (4.96, 5.86)	< 0.001
HbA1c, %	5.95 ± 1.34		5.84 ± 1.33	5.84 ± 1.21	5.92 ± 1.42	0.172
LVM, g	158.22 ± 31.22		155.78 ± 32.28	157.09 ± 32.42	156.45 ± 32.94	0.364
LVMI, g/m^2^	106.83 ± 24.18		106.31 ± 24.47	107.04 ± 25.21	106.58 ± 26.73	0.926

Data were expressed as mean ± SD, median (first quartile, third quartile), or n (%). Lp(a): Q1 ≤ 0.04, 0.04< Q2 ≤ 0.17, 0.17< Q3 ≤ 0.36, Q4 > 0.36. Abbreviation: Lp(a), lipoprotein(a); PIR, poverty income ratio; LVH, left ventricular hypertrophy; BMI, body mass index; SBP, systolic blood pressure; DBP, diastolic blood pressure; TG, triglycerides; TC, total cholesterol; LDL-C, low-density lipoprotein cholesterol; HDL-C, high-density lipoprotein cholesterol; BUN, blood urea nitrogen; CR, creatinine; UA, uric acid; FPG, fasting plasma glucose; HbA1c, hemoglobin A1c; LVM, left ventricular mass; LVMI, left ventricular mass index.

### Association between Lp(a) and LVH

In multivariate logistic regression analyses (**Table 3**), Lp(a) was strongly associated with LVH whether it was used as a continuous variable or a categorical variable when adjusting for age and sex only (per 1-unit increment, OR: 1.516, 95% CI: 1.171-1.963, P = 0.002; Q4 vs Q1, OR: 1.602, 95% CI: 1.274-2.013, P< 0.001; respectively). And in the fully adjusted model, higher Lp(a) was still associated with a higher risk of LVH (per 1-unit increment, OR: 1.366, 95% CI: 1.043-1.789, P = 0.024; Q4 vs Q1, OR: 1.508, 95% CI: 1.185-1.918, P = 0.001; respectively).

**Table 3:** Multivariate logistic regression analysis of the association between Lp(a) and LVH.

	Model 1		Model 2		Model 3		Model 4	
	OR (95% CI)	P value	OR (95% CI)	P value	OR (95% CI)	P value	OR (95% CI)	P value
Q1	Ref	–	Ref	–	Ref	–	Ref	–
Q2	1.255 (0.991, 1.588)	0.059	1.271 (1.003, 1.610)	0.047	1.296 (1.020, 1.648)	0.034	1.294 (1.013, 1.653)	0.039
Q3	1.237 (0.977, 1.567)	0.077	1.239 (0.977, 1.570)	0.077	1.220 (0.960, 1.552)	0.105	1.182 (0.924, 1.512)	0.183
Q4	1.602 (1.274, 2.013)	< 0.001	1.597 (1.269, 2.009)	< 0.001	1.549 (1.226, 1.956)	< 0.001	1.508 (1.185, 1.918)	0.001
P for trend	–	0.001	–	0.001	–	0.003	–	0.008
Lp(a)^a^	1.516 (1.171, 1.963)	0.002	1.492 (1.150, 1.935)	0.003	1.386 (1.065, 1.804)	0.015	1.366 (1.043, 1.789)	0.024

a The OR was examined by per 1-unit increase of Lp(a). Model 1: adjusted for age and sex. Model 2: adjusted for variables included in Model 1 and race, family PIR, ideal exercise, drinking. Model 3: adjusted for variables included in Model 2 and diabetes, hypertension, hypotensive drugs, hypoglycemic drugs. Model 4: adjusted for variables included in Model 3 and BMI, SBP, DBP, TC, BUN, CR, UA, FPG, HbA1c. Abbreviation: Lp(a), lipoprotein(a); LVH, left ventricular hypertrophy; PIR, poverty income ratio; BMI, body mass index; SBP, systolic blood pressure; DBP, diastolic blood pressure; TG, triglycerides; TC, total cholesterol; HDL-C, high-density lipoprotein cholesterol; BUN, blood urea nitrogen; CR, creatinine; UA, uric acid; FPG, fasting plasma glucose; HbA1c, hemoglobin A1c; OR, odd ratio; CI, confidence interval.

**Figure 2:**
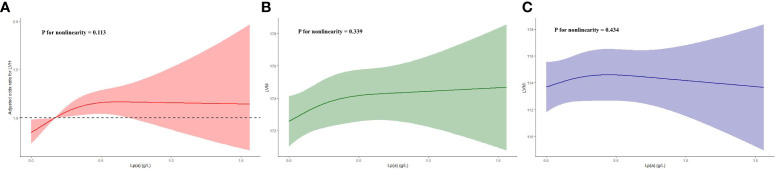
Restricted cubic spline plots of the association between Lp(a) and LVH **(A)**, LVM **(B)** and LVMI **(C)**. Lp(a), lipoprotein(a); LVH, left ventricular hypertrophy; LVM, left ventricular mass; LVMI, left ventricular mass index.

And in the subgroup analysis (**Table 4**), the risk of developing LVH in participants with higher Lp(a) was 1.6, 1.5, 2.5, 1.7, 1.6, and 1.8 times higher than in participants with lower Lp(a) in the subgroups aged< 60 or ≥ 60 years, male, with BMI< 30 kg/m^2^, and with hypertension or without diabetes, respectively (P< 0.05). Additionally, we evaluated LVH according to the Sokolow-Lyon criterion, Cornell criterion and Cornell product methods, respectively, and found that after adjusting for confounding variables, only Lp(a) as a four-categorical variable was strongly associated with the LVH assessed by the Sokolow-Lyon criterion (P< 0.05). However, the correlation of Lp(a) with LVH assessed by the Cornell criterion and Cornell product methods could not be further determined (P > 0.05) (**Table 5**). In addition, we did not find a nonlinear relationship between Lp(a) and LVH, LVM, and LVMI in the RCS analysis (P for nonlinearity > 0.05) (**Figure 2**).

**Table 4:** Subgroups analysis for the associations between Lp(a) and LVH.

	Q1	Q2	Q3	Q4		
	OR	OR (95% CI)	OR (95% CI)	OR (95% CI)	P for trend	
Age						
< 60 years	Ref.	1.253 (0.818, 1.919)	1.090 (0.708, 1.679)	1.554 (1.037, 2.328)*	0.130	
≥ 60 years	Ref.	1.335 (0.986, 1.807)	1.227 (0.906, 1.662)	1.475 (1.089, 1.997)*	0.078	
Sex						
Male	Ref.	1.636 (1.105, 2.423)*	1.608 (1.087, 2.381)*	2.459 (1.668, 3.625)***	< 0.001	
Female	Ref.	1.095 (0.795, 1.509)	0.968 (0.700, 1.337)	1.103 (0.808, 1.507)	0.801	
BMI						
≥ 30 kg/m^2^	Ref.	1.077 (0.702, 1.654)	0.997 (0.651, 1.527)	1.274 (0.836, 1.942)	0.639	
< 30 kg/m^2^	Ref.	1.407 (1.038, 1.908)*	1.288 (0.947, 1.751)	1.685 (1.249, 2.273)**	0.007	
Diabetes						
Yes	Ref.	0.862 (0.548, 1.356)	0.939 (0.605, 1.456)	1.180 (0.762, 1.828)	0.613	
No	Ref.	1.576 (1.167, 2.128)**	1.340 (0.986, 1.822)	1.756 (1.302, 2.369)***	0.002	
Hypertension						
Yes	Ref.	1.310 (0.966, 1.775)	1.202 (0.891, 1.621)	1.599 (1.195, 2.138)**	0.015	
No	Ref.	1.381 (0.910, 2.095)	1.216 (0.781, 1.895)	1.387 (0.892, 2.156)	0.402	

The model used in the subgroups analysis consisted of all covariates used in Model 4 except for the variables that were used for stratification. The OR was examined regarding Q1 as reference. Lp(a), lipoprotein(a); LVH, left ventricular hypertrophy; BMI, body mass index; OR, odd ratio; CI, confidence interval. *p< 0.05, **p< 0.01, ***p< 0.001.

**Table 5:** Association between Lp(a) and LVH assessed by the Sokolow-Lyon criterion, Cornell criterion, and Cornell product, respectively.

		Model 1		Model 2		Model 3		Model 4	
		OR (95% CI)	P value	OR (95% CI)	P value	OR (95% CI)	P value	OR (95% CI)	P value
Sokolow-Lyon criterion	Q1	Ref	-	Ref	-	Ref	-	Ref	-
	Q2	2.716 (1.604, 4.598)	< 0.001	2.416 (1.382, 4.223)	0.002	2.425 (1.385, 4.244)	0.002	2.307 (1.305, 4.078)	0.004
	Q3	3.888 (2.343, 6.450)	< 0.001	2.191 (1.254, 3.829)	0.006	2.188 (1.249, 3.833)	0.006	2.090 (1.181, 3.699)	0.011
	Q4	4.356 (2.638, 7.194)	< 0.001	4.009 (1.144, 3.530)	0.015	1.987 (1.129, 3.496)	0.017	1.870 (1.047, 3.338)	0.034
	P for trend	–	< 0.001	–	0.017	–	0.017	–	0.032
	Lp(a)^a^	2.423 (1.640, 3.578)	< 0.001	0.831 (0.507, 1.361)	0.461	0.810 (0.491, 1.335)	0.408	0.823 (0.478, 1.415)	0.481
Cornell criterion	Q1	Ref	–	Ref	–	Ref	–	Ref	–
	Q2	0.838 (0.547, 1.284)	0.416	0.843 (0.533, 1.333)	0.465	0.865 (0.543, 1.379)	0.543	0.861 (0.535, 1.386)	0.538
	Q3	1.135 (0.764, 1.688)	0.530	1.035 (0.667, 1.606)	0.878	1.072 (0.684, 1.680)	0.763	1.050 (0.660, 1.669)	0.838
	Q4	1.082 (0.728, 1.609)	0.696	0.837 (0.525, 1.334)	0.455	0.795 (0.494, 1.279)	0.344	0.849 (0.517, 1.393)	0.517
Cornell criterion	Q1	Ref	–	Ref	–	Ref	–	Ref	–
	P for trend	–	0.522	–	0.682	–	0.530	–	0.738
	Lp(a)^a^	1.253 (0.793, 1.981)	0.334	0.740 (0.420, 1.303)	0.297	0.685 (0.386, 1.215)	0.196	0.760 (0.415, 1.390)	0.373
Cornell product	Q1	Ref	–	Ref	–	Ref	–	Ref	–
	Q2	0.953 (0.705, 1.289)	0.754	0.986 (0.713, 1.364)	0.933	0.984 (0.709, 1.367)	0.925	0.997 (0.713, 1.393)	0.984
	Q3	1.255 (0.943, 1.670)	0.119	1.173 (0.854, 1.609)	0.324	1.188 (0.863, 1.637)	0.291	1.207 (0.870, 1.676)	0.261
	Q4	1.284 (0.966, 1.707)	0.085	1.113 (0.800, 1.548)	0.526	1.091 (0.780, 1.525)	0.611	1.141 (0.807, 1.614)	0.456
	P for trend	–	0.093	–	0.678	–	0.646	–	0.604
	Lp(a)^a^	1.610 (1.175, 2.207)	0.003	1.215 (0.834, 1.772)	0.310	1.181 (0.807, 1.728)	0.391	1.248 (0.838, 1.857)	0.276

a The OR was examined by per 1-unit increase of Lp(a). Model 1: adjusted for age and sex. Model 2: adjusted for variables included in Model 1 and race, family PIR, ideal exercise, drinking. Model 3: adjusted for variables included in Model 2 and diabetes, hypertension, hypotensive drugs, hypoglycemic drugs. Model 4: adjusted for variables included in Model 3 and BMI, SBP, DBP, TC, BUN, CR, UA, FPG, HbA1c. Abbreviation: Lp(a), lipoprotein(a); LVH, left ventricular hypertrophy; PIR, poverty income ratio; BMI, body mass index; SBP, systolic blood pressure; DBP, diastolic blood pressure; TG, triglycerides; TC, total cholesterol; HDL-C, high-density lipoprotein cholesterol; BUN, blood urea nitrogen; CR, creatinine; UA, uric acid; FPG, fasting plasma glucose; HbA1c, hemoglobin A1c; OR, odd ratio; CI, confidence interval.

## Discussion

In this large population-based cross-sectional observational study, we found that higher Lp(a) was strongly associated with a higher prevalence of LVH assessed by ECG in the general population, and this association was further confirmed to be linear. Additionally, we also demonstrated robustness of the association of Lp(a) with LVH in people aged< 60 or ≥ 60 years, male, with BMI< 30 kg/m^2^, with hypertension or without diabetes.

Current evidence from epidemiological studies suggests that LVH is strongly associated with CVD as well as cardiovascular and all-cause mortality (4, 31–33). Therefore, it is very important to identify the risk factors of LVH and carry out early intervention to prevent the premature occurrence of LVH. Currently, an increasing number of studies have identified independent risk factors for LVH, such as age, hypertension, diabetes and obesity (1, 34, 35). However, to our knowledge, a previous study only showed an association between Lp(a) and LVH assessed by echocardiography in patients with acute myocardial infarction (21), whereas the correlation between Lp(a) and LVH in the general population remains unclear, especially for LVH diagnosed by ECG. Recently, a large observational epidemiological study involving 309,400 participants showed that Lp(a) in the highest tertile was significantly associated with a higher prevalence of LVH diagnosed by echocardiography compared with Lp(a) in the lowest tertile, but not with a higher incidence of LVH during follow-up (36). Although this study demonstrated in the cross-sectional section that participants in the highest tertile of Lp(a) had a 1.3 times higher risk of developing LVH compared with those in the lowest tertile, because LVH in this study was defined by expensive and highly subjective echocardiography, the results may not be applicable to large epidemiological studies based on the general population. Nevertheless, fortunately, our findings were consistent with the conclusion of the above study on the correlation between Lp(a) and LVH, that is, participants in the highest quartile of Lp(a) had a 1.5 times higher risk of suffering from ECG-defined LVH than those in the lowest quartile of Lp(a). Furthermore, in addition to the current evidence confirming the causal association of Lp(a) with atherogenic CVD as well as CAVD (15, 19, 20), several studies have identified other pathogenic phenotypes of Lp(a). For example, Dentali et al. demonstrated in a systematic review and meta-analysis including 14,011 participants and 14 observational studies that higher Lp(a) was significantly associated with higher venous thromboembolism (OR:1.56,95% CI:1.36-1.79) using a random effects model (18). Another observational study showed that although familial hypercholesterolemia did not cause elevated Lp(a), elevated Lp(a) predicted the occurrence of familial hypercholesterolemia (37). However, higher Lp(a) is not always detrimental. For example, Garg et al. found that higher levels of Lp(a) were independently associated with a lower incidence of atrial fibrillation during follow-up in a community-based prospective cohort of 6,593 adults without CVD (17). And Lamina et al. also revealed a negative association of Lp(a) with diabetes (38). However, the above studies only measured the concentration of circulating Lp(a) at baseline, but did not observe the effect of dynamic changes of Lp (a) on CVD. Trinder et al. refined the study design based on the above studies and showed that there was no significant change in the circulating molar concentration of Lp(a) during the median follow-up period of 4.42 years [baseline vs follow-up: 19.50 (7.56-72.50) vs 20.40 (7.70-77.50)], and that Lp(a) at baseline and follow-up were both significantly associated with the occurrence of CVD during follow-up, while changes in Lp(a) had no significant effect on the incidence of CVD (39), indicating that a single measurement of Lp(a) is essential for the primary prevention of CVD in the general population without treatment that significantly changes the level of circulating Lp(a).

Although our study demonstrated the association of Lp(a) with LVH, the mechanisms involved are still unknown. Based on published studies, we proposed the following hypotheses. First, Bergmark et al. used immunoprecipitation and ultracentrifugation experiments, *in vitro* transfer studies and chemiluminescence ELISAs experiments to evaluate the priority of Lp(a) as a carrier of oxidized phospholipids in human plasma. The results showed that most of oxidized phospholipids and Lp(a) co-precipitated in immunoprecipitation experiments, and most of oxidized phospholipids existed in components containing apolipoprotein(a) after subsequent ultracentrifugation experiments, and apolipoprotein(a) was the most important part of Lp(a) structure. Further *in vitro* transfer studies showed that oxidized phospholipids could be preferentially transferred to Lp(a) by oxidized LDL in a time-and temperature-dependent manner regardless of the nature of the buffer. Based on these data, we could draw a conclusion that apolipoprotein(a) or Lp(a) could indirectly participate in oxidative stress as the priority carrier of human plasma oxidized phospholipids, and then activate the markers related to oxidative stress, which might eventually promote the occurrence and development of LVH (40). Second, Aung et al. first conducted a single-sample Mendelian randomized study on 17,311 European individuals from British Biobank, which showed that there was an exact causal relationship between higher LDL and higher LVM, and then conducted a two-sample Mendelian randomized study on another genetic data, which also confirmed this conclusion (41), and since Lp(a) as an LDL-like particle has a cholesterol component that acts similarly to LDL-C (42), we hypothesized that the cholesterol component in Lp(a) plays a role in promoting LVH. Third, a previous study has shown that Lp(a) has a ring structure and inactive protease region similar to plasmin precursor protein structure, which can inhibit fibrinolysis and promote thrombosis through competitive binding of fibrinolytic proteins (43), and Lip et al. conducted a cross-sectional study of 178 patients from hypertension clinic, they estimated LVM, LVMI and LVH by echocardiography, the results showed that hypertensive patients had higher Lp(a) levels and left ventricular septum and posterior wall thickness. Further analysis revealed the correlation between plasma fibrinogen level with homology to Lp(a) and LVM, LVMI and LVH. Therefore, based on these complicated relationships, the hypothesis that Lp(a) indirectly affects left ventricular structure and LVH through fibrinogen or thrombogenic state may also be widely recognized (44). Additionally, current evidence confirms the association of Lp(a) with aortic stenosis and hypertension, and aortic stenosis or hypertension has been proved to be closely related to the left ventricular afterload and occurrence and development of LVH (15, 16).Consequently, we assumed that Lp(a) could promote LVH by causing aortic stenosis and hypertension in the early stages of the disease. In addition to the above findings, we believe that there are still other potential mechanisms that need to be further explored.

Despite the valuable findings, this study still had several limitations. First, we failed to confirm the causal association of Lp(a) with LVH due to the limitations of cross-sectional observational studies. Second, we only studied LVH from ECG sources and did not compare it with LVH detected by echocardiography or cardiac magnetic resonance, and the study population was limited to adults in the United States, so the robustness of the association between Lp(a) and LVH still needs to be further explored. Third, although we controlled for some confounding factors in our study, there may still be other potential risk factors for LVH, such as unhealthy diet and genetic susceptibility. Finally, there are up-to-date criteria for assessing LVH by ECG or echocardiography (45), whereas in this study we used previous criteria for assessing LVH by ECG, so the results might not be representative and more studies are needed to further validate the stability and outreach of the results.

## Conclusions

In this large adult-based observational study, we found that higher Lp(a) levels were significantly associated with a higher risk of developing LVH assessed by ECG in the general population, and we further confirmed the consistency of this association in some specific populations.These findings suggest that early intervention for excessive Lp(a) levels in adults and the development of prevention and treatment measures matched to high-risk populations may help prevent premature onset and excessive prevalence of LVH. Nevertheless, due to several hypothesized causative mechanisms the intervention for excessive Lp(a) levels is due to be further examined in experimental and clinical studies.

## Data availability statement

The original contributions presented in the study are included in the article/supplementary material. Further inquiries can be directed to the corresponding authors.

## Ethics statement

The studies involving humans were approved by National Center for Health Statistics of the Center for Disease Control and Prevention Institutional Review Board. The studies were conducted in accordance with the local legislation and institutional requirements. The participants provided their written informed consent to participate in this study.

## Author contributions

XJY, JG, ZWW and CJQ: Writing – review & editing. JG: Data curation. XJY: Data curation, Writing – original draft. XJY and ZWW: Conceptualization, Methodology, Software. FFW and CJQ: Conceptualization, Funding acquisition, Project administration, Supervision. All authors read and approved the final manuscript.
